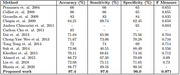# Layer‐wise Adaptive Sine Activation Based Recurrent Network for MCI Conversion

**DOI:** 10.1002/alz70856_096538

**Published:** 2025-12-24

**Authors:** Vishal Deshwal, Arush Jasuja, Harsh Bhasin, Paul Hurley

**Affiliations:** ^1^ International Centre for Neuromorphic Systems (ICNS), Western Sydney Universiy, Sydney, NSW, Australia; ^2^ New York University, New York, NY, USA; ^3^ Bennett University, Greater Noids, Uttar Prdesh, India; ^4^ Western Sydney University, Sydney, NSW, Australia

## Abstract

**Background:**

Mild Cognitive Impairment (MCI) can be considered as one of the early markers of dementia and can be helpful for clinicians to take corrective measures and delay its progression. This study aims to classify Mild Cognitive Impairment‐Converts (MCI‐C) and Mild Cognitive Impairment‐Non‐Converts (MCI‐NC) using structural Magnetic Resonance Imaging (s‐MRI) by analysing the decay in gray matter using a novel approach. Previous works such as 2D or 3D CNNs had drawbacks: 2D CNNs cannot detect spatial correlation between MRI slices, while 3D CNNs are computationally expensive and less practical to use on edge devices.

**Method:**

To overcome these challenges, we propose a novel sequence‐based framework inspired by Natural Language Processing (NLP) techniques, designed to capture correlations between MRI slices. The study used s‐MRI volumes from 187 subjects (75 MCI converters, 112 MCI non‐converters) obtained from the Alzheimer's Disease Neuroimaging Initiative (ADNI), retaining 106 slices per volume. For each slice, histograms were created using the local binary pattern (LBP) and its variants (Basic LBP, Uniform LBP, and Rotation‐Invariant Uniform LBP), reducing the dimensionality and forming feature vectors. These feature vectors were stacked for each MRI volume, creating a train of features. For the classification model, we used a Layer‐wise Adaptive Sine Activation (LASA) based Bidirectional Recurrent Neural Network (BiRNN) capable of modelling the temporal and spatial relationships in the data. The trainable frequency parameter in LASA enables the network to adapt both short‐term and long‐term dependencies, while the bidirectional structure captures forward and backward correlations between slices.

**Result:**

Throughout training, the validation accuracy consistently exceeded the accuracy of the training, indicating a strong generalisation performance. Across 30 experiments, our model achieved an average accuracy of 97.4% with a standard deviation of ±0.2, demonstrating its effectiveness and reliability.

**Conclusion:**

The high accuracy and edge‐device compatibility of this method have the potential to significantly improve clinical practice by allowing early and cost‐effective diagnosis of MCI in a wider range of healthcare settings, including remote and resource‐constrained environments. This innovative method offers an efficient and practical solution for the early diagnosis of dementia, overcoming the limitations of traditional deep learning based models.